# Clinical values of Ku80 upregulation in superficial esophageal squamous cell carcinoma

**DOI:** 10.1002/cam4.1314

**Published:** 2018-03-13

**Authors:** Shuai Wang, Junjie Xi, Zongwu Lin, Jiatao Hao, Can Yao, Cheng Zhan, Wei Jiang, Yu Shi, Qun Wang

**Affiliations:** ^1^ Department of Thoracic Surgery Zhongshan Hospital Fudan University Shanghai China; ^2^ General Practice Department Zhongshan Hospital Fudan University Shanghai China; ^3^ Department of Gastroenterology Zhongshan Hospital Fudan University Shanghai China

**Keywords:** DNA repair, Esophageal cancer, prognosis Ku80, survival

## Abstract

Ku80 is an important DNA repair protein. Here, this study sought to investigate clinical impacts of Ku80 expression for patients with superficial esophageal squamous cell carcinoma (ESCC). Immunohistochemical analysis of Ku80 expression was carried out in normal esophageal mucosa, squamous epithelial dysplasia, carcinoma in situ, and superficial ESCC. Its relationships with clinicopathological features and survival of superficial ESCC patients were further clarified. Lentivirus‐mediated RNA interference was used to silence Ku80 gene in ECA109 and KYSE150 cells. Both quantitative real‐time PCR and Western blot were employed to evaluate Ku80 levels. CCK‐8 assay, clone formation assay, flow cytometry, and tumorigenesis experiment were performed to evaluate the malignant phenotype of ECA109 and KYSE150 cells. Increased Ku80 expression was observed in dysplastic esophageal mucosa and carcinoma in situ compared to normal esophageal mucosa (*P *< 0.001, *P *< 0.001). Ku80 expression was further increased in superficial ESCC in comparison with dysplastic esophageal mucosa and carcinoma in situ (*P *< 0.001, *P *= 0.034). In superficial ESCC, Ku80 overexpression was related to tumor differentiation (*P *= 0.017), T status (*P *= 0.011), nodal involvement (*P *= 0.005), TNM stage (*P *= 0.004), and postoperative recurrence (*P *= 0.008). Cox proportional hazards regression showed tumor differentiation, T status, nodal involvement, TNM stage, and Ku80 expression were both independent predictors of patients’ overall survival and disease‐free survival. Ku80 shRNA effectively reduced Ku80 expression, which significantly inhibited proliferation, clone formation, and induced apoptosis in ECA109 and KYSE150 cells. The tumor growth of xenografts was significantly reduced by Ku80 silencing in ECA109 and KYSE150 cells. Ku80 overexpression associates with unfavorable prognosis of superficial ESCC patients, and silencing of Ku80 could inhibit the malignant behavior of ESCC cells. We provide evidence that Ku80 has unrecognized roles in carcinogenesis and development of ESCC.

## Introduction

Esophageal cancer accounts for 3.8% of the overall malignant tumors and 5.4% of the mortality in the world 1, while esophageal squamous cell carcinoma (ESCC) is the most common histological types of esophageal carcinomas in East Asia 2. Due to insidious symptomatology, local invasiveness, and remote metastases, ESCC is considered as a highly aggressive malignant tumor.

Despite improvements in multimodality therapies, the 5‐year survival rate for ESCC remains 15–24% 3. Surgery is the first management choice for patients with resectable ESCC. However, the therapeutic efficiency is not satisfactory and about half of patients would relapse within 1–3 years after esophagectomy 4. According to Japanese Society for Esophageal Diseases, superficial ESCC is considered as lesion invading the mucosa or submucosa layer and less than muscularis propria regardless of nodal metastasis 5. Patients with superficial ESCC can be treated with radical surgery or endoscopic resection 6. Based on NCCN guideline, patients in early stage accepting radical resection of tumor do not need postoperative adjuvant therapy. But our previous study indicated even en bloc resection of tumor and lymph node was achieved, and some patients with superficial ESCC experience nodal involvement after surgery 7. In our opinion, it is necessary to stratify superficial ESCC according to different clinical outcomes. Prognosis of ESCC is tumor staging specific, but the accuracy and sensitivity need to be improved 8, 9. Therefore, it is paramount and urgent to explore tumor biomarkers to predict superficial patients’ survival and recurrence. Previous reports have attempted to study molecular predictors of outcomes in ESCC 10, 11. However, no tumor biomarker has been applied popularly in ESCC. So, specific and reliable biomarkers are still necessary.

ESCC is featured with genome instability resulting from serious DNA damage because of genetic or epigenetic factors 12. DNA damage drives carcinogenesis of ESCC 12, 13, 14. Ku80, also known as Homo sapiens X‐ray repair complementing defective repair in Chinese hamster cells 5 (XRCC5), is an important and specific components of nonhomologous end joining (NHEJ) 15. Ku80 could bind to broken ends of DNA, initiate NHEJ repair, and remove double‐strand break (DSB). Ku80 is also involved in multiple cellular behavior, such as cell cycle, telomere maintenance, proliferation, and apoptosis 16. It is worth noting that upregulation of Ku80 occurs in diverse malignant tumors, such as bladder cancer, gastric cancer, colorectal cancer, and breast cancer 17, 18, 19, 20. Our previous studies indicated evaluation of Ku80 promotes management and stratification of ESCC patients 21, 22. However, no study has evaluated clinical values of Ku80 in superficial ESCC.

In this study, we concentrated on clinical significance of Ku80 in superficial ESCC. We investigated the association between Ku80 level and clinical features and prognosis of superficial ESCC. To confirm our clinical findings, we detected effect of lentivirus‐mediated Ku80 knockdown in ESCC cells and evaluated the potential of Ku80 as a therapeutic target.

## Materials and Methods

### Patients, tissues, and cells

This study was approved by Research Ethics Committee of Zhongshan Hospital, Fudan University. Written informed consent was obtained for collection and detection of samples, anonymous data analysis, and manuscript publication. The informed consent included statements as follows: (1) All participants provided their formal consent to participating in the research process; (2) information on researcher’ roles, research processes, and individual examination results was provided; (3) all participants know the inclusion and exclusion criteria and were free to withdraw; and (4) the final thesis and possible significant elements of the project will be published; however, no individual respondents will be identified or identifiable. From May 2010 through July 2011, the first cohort of 107 midthoracic superficial ESCC patients (20 women and 87 men, mean age: 45.8 ± 9.3 years) who underwent Ivor Lewis esophagectomy were screened in our hospital. Detail surgery process was reported in our previous studies 7, 10, 23. The inclusion standards were as follows: (1) Ivor Lewis esophagogastrectomy with two‐field nodal resection was performed, according to 2009 Union for International Cancer Control (UICC) 24. The tumor diagnosis was confirmed by histopathological detection, based on criteria established by Japanese Society for Esophageal Diseases 5. Pathological examination showed en bloc resection of malignant tumor. (2) Patients did not receive chemotherapy, radiotherapy, or biotherapy before tissues collection. (3) Patients who died perioperatively were excluded. Full medical documents were collected, and clinicopathologic characteristics were analyzed. Tumors were restaged according to the 2009 UICC TNM staging guidelines 24.

Meanwhile, the second cohort of patients was collected from the Department of Gastroenterology, Zhongshan Hospital, Fudan University. For these patients, 83 patients (14 women and 69 men, mean age: 41.7 ± 10.9 years) were esophageal squamous epithelial dysplasia (including 36 mild and 47 moderate dysplasia) and 65 squamous cell carcinoma in situ (11 women and 54 men, mean age: 41.4 ± 9.7 years). These patients who underwent endoscopic resection were confirmed via pathological examination after resection 5. In control group, 71 volunteers (25 women and 46 men, mean age: 45.6 ± 11.2 years) without any malignant disease were selected from General Practice Department, Zhongshan Hospital, Fudan University. The inclusion standards of volunteers were as follows: (1) Normal esophageal mucosa was confirmed by histopathology examination. (2) Samples were acquired by electronic gastroscopy, which indicated no sign of inflammation, hyperemia, ischemia, or abnormal mass. (3) Enhanced CT scan showed the absence of esophageal mass or mediastinal enlarged lymph nodes. (4) Individuals did not have abnormal symptoms, such as eructation, acid regurgitation, heartburn, dysphagia, and obstruction of esophagus. Several protective measures are taken for health control volunteers who were collected esophageal mucosa samples. We provide participants with a very brief overview of the project and where they can access further information. They will be given the opportunity to withdraw from the research at any time. None of them are considered to be “vulnerable.” Tissues were collected with small size of 0.5 × 0.3 × 0.3 cm. Samples collections were performed only once for individual participant by electronic gastroscopy. Results of electronic gastroscopy were freely accessible for participant, and copies of a summary final report would be provided. The data were stored in locked cabinets protected with the highest security software, with reference to the data protection legislation. The individual information would not be available to others. Subjects with drug, smoking, and alcohol abuse were excluded. Individuals with history of malignant disease were also excluded. There is no consanguineous relationship among these individuals, and all of them were Han people in China. The questionnaire collected individual information, and there are no significant differences within superficial ESCC group, esophageal squamous epithelial dysplasia group, squamous cell carcinoma in situ group, and control group (e.g., age, gender, family history, medications, and individual exposure to carcinogens).

The degeneration tissue and corresponding healthy esophageal mucosa (CHEM) were harvested from each patient. The CHEM was harvested at a distance of more than 5 cm from margin of degeneration tissue. Seventy‐one normal esophageal mucosal (NEM) tissues in control group were harvested by gastroscopy. No sign of deterioration and necrosis was found in CHEM and NEM by microscope examination. All samples were rinsed in cold 0.9% NaCl and stored at ‐80°C.

The human ESCC ECA109 and KYSE150 cells (purchased from the Institute of Cytobiology, Chinese Academy of Sciences) were cultured in RPMI‐1640 containing 10% heat‐inactivated fetal calf serum, 100 U/mL of penicillin, and 100 *μ*g/mL of streptomycin at 37°C in a humidified incubator with 5% CO_2_.

### Immunohistochemistry

As reported in our previous study, immunohistochemistry (IHC) was performed by UltraVision Quanto Detection System (Thermo Scientific, Fremont, CA, USA) 22. Sections were cultured in hydrogen peroxide block. After incubation with UltraVision Protein Block and the rabbit monoclonal anti‐human Ku80 antibody (1:500; Abcam Ltd., Cambridge, UK) overnight at 4°C, specimens were cultured in primary antibody amplifier Quanto. Then, the specimens were cultured in HRP polymer Quanto and 3,3‐diaminobenzidine. HeLa cells were used to validate specificity of antibody 25. The immunohistochemical scoring (IHS) was calculated according to a semiquantitative system 22.

### ROC curve

MedCalc statistical software package 13.0.2.0 (MedCalc Software bvba, Belgium) was used to evaluate the data of Ku80 level. The threshold value for Ku80 level was screened by receiver operating characteristics (ROC). Cutoff value was defined as the IHS of Ku80 with optimal sensitivity and specificity.

### Follow‐Up

After Ivor Lewis esophagectomy, superficial ESCC patients were reexamined regularly (included physical examination, CT, ultrasound, and some patients received PET scan, and biopsy). Regional nodal enlargement was diagnosed as postoperative nodal recurrence. After exclusion of primary malignancy, ESCC occurred in the remote organs was diagnosed as metastatic tumor. The overall survival (OS) time and disease‐free survival (DFS) time were defined as reported in our previous studies 21, 22. The follow‐up was ended in August 2016.

### Lentivirus‐mediated short hairpin RNA (shRNA)

GV115 vectors targeting Ku80 and nonsilencing vector were obtained from Shanghai GeneChem. CO., LTD (Shanghai, China). GV115 cloning vector included elements for expression packaging construct into virions and GFP reporter. The scramble sequences were 5′‐TTCTCCGAACGTGTCACGT‐3′. The Ku80 shRNA included shRNA1, shRNA2, and shRNA3: 5′‐CTTTAACAACTTCCTGAAA‐3′(shRNA1), 5′‐TGCAATTCTTCTTGCCTTT‐3′ (shRNA2), and 5′‐TCATATCAAGCATAACTAT‐3′ (shRNA3). Translentiviral packaging mix shRNA transfer vector was cotransfected into HEK293T packaging cells (Shanghai GeneChem. CO., LTD). For cell infection, ECA109 and KYSE150 cells were incubated with viral supernatants and polybrene (6 *μ*g/mL) for 12 h. Cells were transduced by the lentiviral particles based on multiplicity of infection. After co‐incubation with lentiviral particles, cells were cultured in fresh complete medium for 72 h. Lentiviral‐mediated transfection efficiency was confirmed by inverted phase contrast microscope. ECA109 and KYSE150 cells were transduced by the lentiviral particles followed by puromycin selection (1 *μ*g/mL) for 10 days. The stable transfected cells were maintained in complete medium with puromycin (0.2 *μ*g/mL). Stability and effectiveness of Ku80 silencing were validated by quantitative real‐time RT‐PCR (qRT‐PCR) and Western blot.

### qRT‐PCR

Tissue RNA was harvested, and reverse transcriptase was performed using M‐MLV reverse transcriptase system (Takara Bio, Inc., Shiga, Japan). The primer sequences of Ku80 (Takara Shuzo Co., Kyoto, Japan) were 5′‐TGACTTCCTGGATGCACTAATCGT‐3′(sense); 5′‐TTGGAG CCAATGGTCAGTCG‐3′ (anti‐sense). The housekeeping gene *β*‐actin (sense: 5′‐GGCGGCACCATGTACCCT‐3′; anti‐sense: 5′‐AGGGGCCGGACTCGTCATACT‐3′) was used to normalize RNA quantity and quality as reference. LightCycler 480 (Roche Diagnostics, Nutley, NJ) was used for PCRs (30 sec at 95°C followed by 45 cycles of 30 sec at 94°C, 60 sec at 30°C, and 30 sec at 72°C). PCRs were terminated at 4°C, following 7‐min elongation at 72°C. The mRNA expression was analyzed as the calibrator normalized ratio using LightCycler 480 software 1.5 (Roche Diagnostics, Nutley, NJ).

### Western blot

Protein extraction, solvation, electrophoresis, and transfer were performed as reported previously 22. Nitrocellulose membranes were cultured in Ku80 or *β*‐actin primary antibodies (1:1,000 dilution; Abcam, MA) overnight at 4°C. Then, membranes were incubated with secondary antibody conjugated with horseradish peroxidase anti‐rabbit IgG (1:10,000; Santa Cruz Biotechnology, Inc.). The protein levels were evaluated by enhanced chemiluminescence detection system (LAS 4000 mini system; General Electric, Fairfield, CT).

### Cell Counting Kit‐8 (CCK‐8) assay

Cells were seeded in 96‐well microtiter plates with 1 x 10^4^ cells/well in a final volume of 100 *μ*L. CCK‐8 (10 *μ*L) was added at 6 h, 12 h, 24 h, 48 h, or 72 h, respectively, and cells were further incubated for 2 h at 37°C. Optical density (OD) was analyzed by microplate reader (SpectraMax M2; Molecular Devices, Sunnyvale, CA) at 450 nm.

### Clone formation assay

Cells were seeded in six‐well plates (BD Biosciences, Franklin Lakes, NJ) and incubated for 2 weeks. After wash, cells were fixed by 4% paraformaldehyde for 20 min. The 0.1% crustal violet was used to stain cells for 10 min. A number of clones with more than 50 cells were detected under microscope.

### Flow cytometry

Cells were seeded into six‐well plates at 3 × 10^5^ cells/well and cultured. After wash with cold PBS, cells were detached by 0.05% trypsin. After three‐time wash, cells were resuspended and stained through the Annexin V‐FITC Apoptosis Detection Kit (Becton Dickinson, San Jose, CA). Samples were examined by a FACScan flow cytometer (BD LSRFortessa^TM^ system, Becton Dickinson).

### Nude mouse xenograft model

A total of 60 4‐week‐old BALB/c mice at 16–18 g (30 females and 30 males) were obtained from Vital River Co. The 60 mice were classified into four groups: (1) blank control group: mice were injected with medium; (2) negative control group: mice were injected with ESCC cells; and (3) two experimental groups: mice were injected with nonsilencing shRNA‐ or Ku80 shRNA‐transfected ESCC cells. BALB/c mice were cultured in specific pathogen‐free facilities. The 1 × 10^6^ cells were subcutaneously injected into the right shoulder of mice. Tumor volumes (*V*) were evaluated as formula: V* *= *π*/6 × width^2^ (mm^2^) × length (mm). The experiment was ended based on institutionally approved guidelines. Inhibition rate (IR) of xenografts was analyzed as formula: IR* *=  (tumor weight in normal control group ‐ tumor weight in experimental group)/tumor weight in normal control group×100%.

### Statistical analysis

Differences in continuous variables were analyzed by one‐way analysis of variance. Statistical comparisons of discontinuous variables in different groups were performed by Mann–Whitney *U* test. Chi‐square test was performed to clarify associations between categorical variables. Survival curves were calculated using the Kaplan–Meier method. Univariate log‐rank test and Cox regression model analysis were performed to identify prognostic significance. A significant difference was defined as a two‐tailed *P*‐value of less than 0.05. All statistical analysis was performed using SPSS.17.0 software (SPSS, Chicago, IL).

## Results

### Superficial ESCC patients and recurrence

The clinical data from the 107 patients with superficial ESCC were shown in Table 1. In this study group, six patients were died due to cardio‐cerebrovascular disease or accident. In other 101 patients, 57 patients (56.4%) had recurrence during follow‐up period. Recurrence patterns consisted of regional recurrence (32/101, 31.7%), remote metastasis (14/101, 13.9%), and combination of regional recurrence and remote metastasis (11/93, 10.9%).

**Table 1:** Correlation of Ku80 expression with clinicopathologic features of superficial ESCC patients

Characteristics	Cases (107)	Ku80 expression level		*P*
		Low (43)	High (64)	
Age (yr)				
≥50	39	18	21	0.420
<50	68	26	43	
Gender				
Male	87	39	49	0.075
Female	20	4	16	
Differentiation degree				
Low	45	12	33	0.017
Mid‐high	62	31	31	
T status				
T1a	53	28	25	0.011
T1b	54	15	39	
Lymph node metastasis				
Yes	31	6	25	0.005
No	76	37	39	
TNM stage				
IA	42	24	18	0.004
IB + IIB + IIIA	65	19	46	
Recurrence^a^				
Yes	57	16	41	0.008
No	44	24	20	

a Six patients who died due to cardio‐cerebrovascular disease or accident were censored (three cases of high Ku80 expression patients and three cases of low Ku80 expression patients). Statistical analysis was performed using the Fisher's exact test. *P*‐values < 0.05 were considered significant.

### Ku80 protein expression profile

**Figure 1:**
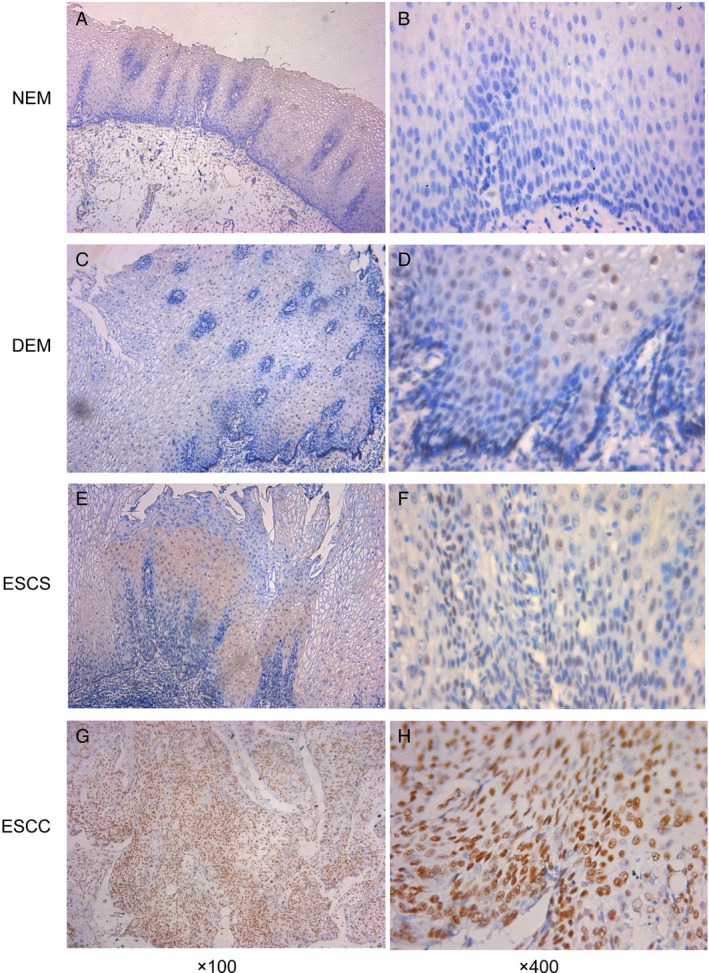
Immunohistochemical staining of Ku80 in esophageal tissues. (A, B) Representative negative expression of Ku80 in normal esophageal mucosa (NEM). (C, D) Representative low expression of Ku80 in dysplastic esophageal mucosa (DEM). (E, F) Representative low expression of Ku80 in esophageal squamous carcinoma in situ (ESCS). (G, H) Representative high expression of Ku80 in superficial esophageal squamous cell carcinoma (ESCC).

**Figure 2:**
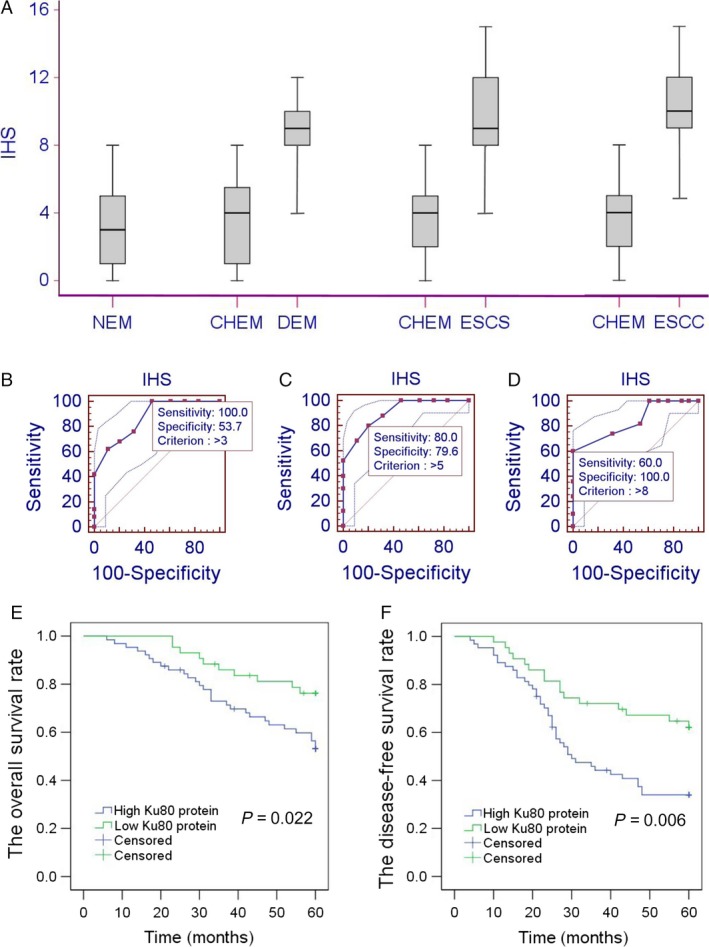
Evaluation of Ku80 as a diagnostic and prognostic marker in superficial ESCC. (A) The immunohistochemical scores (IHS) of Ku80 in normal esophageal mucosa (NEM), dysplastic esophageal mucosa (DEM), esophageal squamous carcinoma in situ (ESCS), superficial ESCC, and their corresponding healthy esophageal mucosa (CHEM). Data are represented as a box‐and‐whisker plot and analyzed using Mann–Whitney U test. ROC analyses of Ku80 protein expression and the selection of cutoff score for DEM (B), ESCS (C), and ESCC (D). (E) High Ku80 expression was significantly associated with reduced overall survival. (F) High Ku80 protein expression was significantly associated with decreased disease‐free survival.

The expression of Ku80 was analyzed in 71 cases of NEM, 83 cases of dysplastic esophageal mucosa (DEM), 65 cases of esophageal squamous carcinoma in situ (ESCS), 107 cases of superficial ESCC, and their CHEM. Positive Ku80 expression was defined as brownish‐yellow stain in nucleus (Fig. 1). The IHS of Ku80 in different tissues was showed in Figure 2A. By Mann–Whitney *U* test, IHS of Ku80 was significantly increased in DEM, ESCS, and ESCC compared with NEM (*P *= 0.001, *P *< 0.001, *P *< 0.001, respectively). Additionally, there was no significant difference between NEM and CHEM. ESCC exhibited the greatest expression level of the Ku80, whose average IHS was significantly higher than DEM and ESCS (*P *< 0.001 and *P *= 0.034). However, difference between DEM and ESCS was not statistically significant (*P *= 0.139).

### Ku80 as a potential diagnostic marker

ROC was performed to distinguish DEM, ESCS, and ESCC from NEM. Based on ROC curve, IHS of 3, 5, and 8 was the point with optimal sensitivity and specificity for DEM, ESCS, and ESCC, respectively, and set as threshold value (Fig. 2B). The area under the ROC curve (AUC) was 0.841 (95% CI: 0.756–0.905, *P *< 0.001), 0.865 (95% CI: 0.784–0.924, *P *< 0.001), and 0.905 (95% CI: 0.831–0.954, *P *< 0.001) for DEM, ESCS, and ESCC, respectively.

### Clinicopathological value of Ku80

In our study, ESCC samples with the IHS greater than or equal to 10 were defined as high Ku80 expression. Superficial ESCC patients were thereby classified into two groups, namely Ku80 high‐level group (*n *= 64, 59.8%) and Ku80 low‐level group (*n *= 43, 40.2%). As showed in Table 1, Chi‐square test suggested Ku80 expression level was associated with differentiation degree (*P *= 0.017), T status (*P *=  0.011), nodal involvement (*P *= 0.005), TNM stage (*P *= 0.004), and tumor recurrence (*P *= 0.008), and not with age (*P *= 0.420) and gender (*P *= 0.075).

### Ku80 expression and survival of superficial ESCC patients

The 1‐, 3‐, and 5‐year OS rates of superficial ESCC were 97.2%, 78.2%, and 62.4%, respectively. For Ku80 low‐level group, the 1‐, 3‐, and 5‐year OS rates were 100.0%, 86.0%, and 76.2%. However, for Ku80 high level, the 1‐, 3‐, and 5‐year OS rates were 84.3%, 72.9%, and 53.1% (Fig. 2E). Univariate analyses indicated differentiation degree (*P *= 0.001), T status (*P *= 0.001), lymph node metastasis (*P *< 0.001), TNM stage (*P *< 0.001), and Ku80 level (*P *= 0.022) were significant prognostic indicators (Table 2). But age (*P *= 0.405) and gender (*P *= 0.066) did not reach the statistical significance. Multivariate analysis suggested tumor differentiation (*P *= 0.013), T status (*P *= 0.004), nodal involvement (*P *= 0.011), TNM stage (*P *= 0.013), and Ku80 level (*P *= 0.016) were both independent predictors of OS.

**Table 2:** Univariate and multivariate analyses of overall survival for superficial ESCC patients

Variable	Univariate analysis			Multivariate analysis		
	HR	95% CI	*P*	HR	95% CI	*P*
Age						
≥50 vs. <50 yrs	1.335	0.676–2.636	0.405			
Gender						
Male vs. Female	1.923	0.957–3.867	0.066			
Differentiation degree						
Low vs. Mid–high	0.313	0.162–0.603	0.001	0.430	0.220–0.840	0.013
T status						
T1a vs. T1b	0.285	0.141–0.574	<0.001	0.343	0.166–0.711	0.004
Lymph node metastasis						
Yes vs. No	0.193	0.102–0.365	<0.001	0.374	0.175–0.797	0.011
TNM stage						
IA vs. IB + IIB + IIIA	0.162	0.063–0.415	<0.001	0.264	0.092–0.760	0.013
Ku80 protein level						
Low vs. High	0.433	0.211–0.888	0.022	0.361	0.158–0.825	0.016

Statistical analysis was performed using the proportional hazard model (Cox). Data considered significant (*P *< 0.05) in the univariate analyses were examined in the multivariate analyses. HR, hazard ratio; CI, confidence interval.

The 1‐, 3‐, and 5‐year DFS rates of superficial ESCC were 92.5%, 55.6%, and 45.5%, respectively. For Ku80 low group, the 1‐, 3‐, and 5‐year DFS rates were 97.7%, 72.1%, and 62.1%, while for Ku80 high group, the 1‐, 3‐, and 5‐year DFS rates were 89.1%, 44.2%, and 34.0% (Fig. 2F). In the 101 patients with complete 5‐year follow‐up data, recurrences were observed in 41 of 61 patients (67.2%) with high Ku80 expression and 16 of 40 (40.0%) patients with low Ku80 expression. Local recurrence rate in high Ku80 expression group (24/61, 39.3%) was obviously higher than that in low Ku80 expression group (8/40, 20.0%). The distant metastases rate in high Ku80 expression group (10/61, 16.4%) was also higher than that in low Ku80 expression group (4/40, 10.0%). Univariate analysis suggested tumor differentiation (*P *< 0.001), T status (*P *= 0.030), nodal involvement (*P *< 0.001), TNM stage (*P *< 0.001), and Ku80 level (*P *= 0.006) were significant prognostic factors (Table 3). Multivariate analyses indicated that tumor differentiation (*P *= 0.023), T status (*P *= 0.003), nodal involvement (*P *= 0.006), TNM stage (*P *= 0.005), and Ku80 expression (*P *= 0.014) were both independent significant indicators of DFS.

**Table 3:** Univariate and multivariate analyses of disease‐free survival for superficial ESCC patients

Variable	Univariate analysis			Multivariate analysis		
	HR	95% CI	*P*	HR	95% CI	*P*
Age						
≥50 vs. <50 years	1.503	0.852–2.653	0.160			
Gender						
Male vs. Female	1.495	0.817–2.735	0.192			
Differentiation degree						
Low vs. Mid–high	0.306	0.178–0.524	<0.001	0.495	0.271–0.907	0.023
T status						
T1a vs. T1b	0.557	0.329–0.944	0.030	0.440	0.253–0.762	0.003
Lymph node metastasis						
Yes vs. No	0.250	0.147–0.424	<0.001	0.438	0.243–0.787	0.006
TNM stage						
IA vs. IB + IIB + IIIA	0.327	0.178–0.599	<0.001	0.396	0.208–0.754	0.005
Ku80 protein level						
Low vs. High	0.443	0.248–0.791	0.006	0.478	0.266– 0.860	0.014

Statistical analysis was performed using the proportional hazard model (Cox). Data considered significant (*P *< 0.05) in the univariate analyses were examined in the multivariate analyses. HR, hazard ratio; CI, confidence interval.

### Stable silencing of oncogenic Ku80 expression

**Figure 3:**
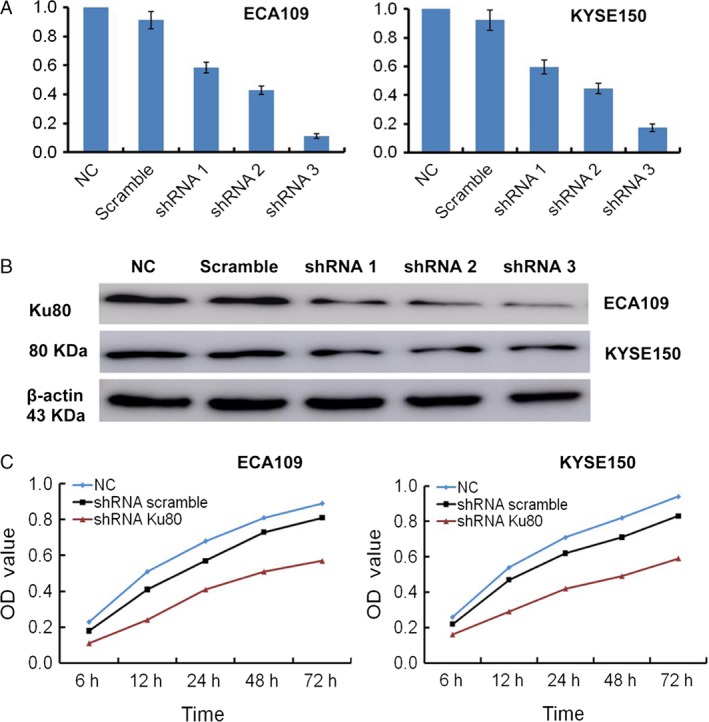
Ku80 silencing inhibited cell proliferation in vitro. (A) The levels of Ku80 were detected by qRT‐PCR. (B) The gel is representative of three independent Western blot assays. (C) Cell proliferation was suppressed by Ku80 knockdown detected by CCK‐8 assay.

To further explore the roles of Ku80 in ESCC, we infected ECA109 and KYSE150 cells with shRNA scramble, shRNA‐1, shRNA‐2, and shRNA‐3. Relative Ku80 mRNA expression in untransfected and transfected shRNA scramble, shRNA‐1, shRNA‐2, and shRNA‐3 ESCC cells was shown in Figure 3A. Western blot assays indicated relative Ku80 protein expression levels were 0.845 ± 0.088, 0.713 ± 0.083, 0.427 ± 0.053, 0.316 ± 0.041, and 0.198 ± 0.03 in untransfected and transfected shRNA scramble, shRNA‐1, shRNA‐2, and shRNA‐3 ECA109 cells (Fig. 3B). Ku80 protein expression levels were 0.823 ± 0.091, 0.745 ± 0.069, 0.448 ± 0.053, 0.326 ± 0.047, and 0.182 ± 0.027 in untransfected and transfected shRNA scramble, shRNA‐1, shRNA‐2, and shRNA‐3 KYSE150 cells. The shRNA‐1, shRNA‐2, and shRNA‐3 could suppress the Ku80 mRNA and protein expression both in ECA109 and KYSE150 cells, whereas shRNA‐3 had the highest efficiency in Ku80 silencing confirmed by qRT‐PCR and Western blot assays. Therefore, we selected Ku80 shRNA‐3 to effectively and specifically knockdown Ku80 gene in further function analyses.

### Depletion of Ku80 inhibits malignancy of ESCC cells in vitro

**Figure 4:**
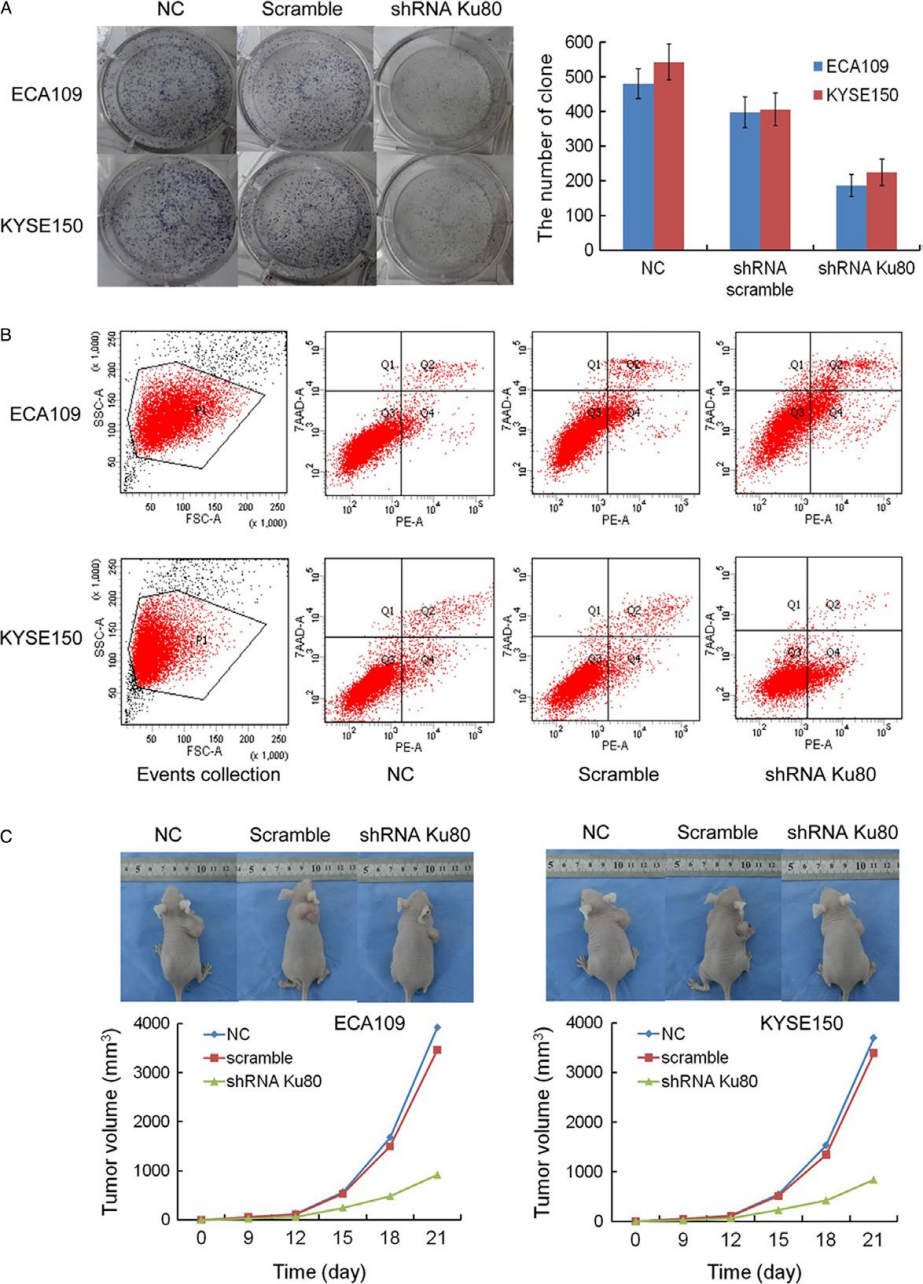
Ku80 silencing inhibited malignant behavior of ESCC in vitro and in vivo. (A) Cell clone formation in vitro was suppressed by Ku80 knockdown. (B) Ku80 silencing induced apoptosis of ESCC cells in vitro. (C) The tumorigenesis of ESCC cells in vivo was significantly inhibited by Ku80 knockdown. Representative photographs of the nude mice at 21 days after injection were shown. Tumor volumes were presented as growth curves.

CCK‐8 assays were applied to examine cell proliferation in vitro. As shown in Figure 3C, the proliferation ability of shRNA Ku80‐transfected ECA109 and KYSE150 cells was decreased obviously compared to shRNA scramble‐transfected cells. The cell proliferation of shRNA scramble‐transfected ECA109 and KYSE150 had no obvious difference with nontransfected cells. In colony‐forming experiment, the number of clones of nontransfected and transfected shRNA scramble, shRNA Ku80 ESCC cells was shown in Figure 4A. The difference between nontransfected ECA109 and KYSE150 cells and shRNA Ku80‐transfected cells have statistical significance (ANOVA; *P *< 0.001, *P *< 0.001). In addition, we applied Annexin V/PI staining to detect apoptosis (Figure 4B). The percentage of apoptosis in nontransfected and transfected shRNA scramble, shRNA Ku80 ECA109 cells were 1.7 ± 0.2, 3.9 ± 0.4, 15.8 ± 1.6. The percentage of apoptosis in nontransfected and transfected shRNA scramble, shRNA Ku80 KYSE150 cells were 1.6 ± 0.3, 2.8 ± 0.4, 14.9 ± 1.5. Flow cytometric analyses showed that Ku80 gene silencing promoted the apoptosis of both cell lines.

### Knockdown of Ku80 inhibits the growth of xenografts in vivo

Xenograft tumor experiment showed that the volumes of nontransfected and transfected shRNA scramble, shRNA Ku80 ECA109 xenograft tumor were 3923 ± 418 mm^3^, 3462 ± 357 mm^3^, 923 ± 89 mm^3^. The volumes of nontransfected and transfected shRNA scramble, shRNA Ku80 KYSE150 xenograft tumor were 3698 ± 384 mm^3^, 3395 ± 341 mm^3^, 846 ± 74 mm^3^. Compared to the nontransfected cells, the volumes of shRNA Ku80‐transfected ECA109 and KYSE150 xenograft tumor were decreased significantly (ANOVA; *P *< 0.001, *P *< 0.001). The weights of xenografts were 3.45 ± 0.41 g, 3.18 ± 0.35 g, 1.09 ± 0.12 g for nontransfected and transfected shRNA scramble, shRNA Ku80 ECA109 cells. The weights of xenografts were 3.07 ± 0.34 g, 2.89 ± 0.31 g, 0.98 ± 0.10 g for nontransfected and transfected shRNA scramble, shRNA Ku80 KYSE150 cells. IRs of tumor xenografts in shRNA Ku80 ECA109 and KYSE150 cells were 68.4% and 68.1%, respectively (Fig. 4C).

## Discussion

Ku80 is a key component of DNA repair proteins 12, 15. Additionally, earlier studies have demonstrated that Ku80 is highly expressed and correlates with the progression of gastric cancer, breast cancer, bladder cancer, and colorectal cancer 17, 18, 19, 20. Our previous studies also reported Ku80 was aberrantly upregulated in ESCC 21, 22. However, the clinical values of Ku80 expression in superficial ESCC are currently unclear.

In this study, we found significant increase in Ku80 expression in DEM, ESCS, and ESCC compared with NEM (Fig. 1). Our salient findings are important in view of the fact that studies on molecular alternations of esophageal dysplasia and carcinoma in situ are limited. Due to lack of serious clinical symptoms, these patients would neglect the preneoplastic lesions and avoid endoscopic examination. In addition, there is no reliable biomarker that can be used in clinics routinely in preneoplastic lesions. Our ROC analyses suggested Ku80 would act as a potential diagnostic marker. Hence, overexpression of Ku80 observed in early stage of disease is an important finding. The expression of Ku80 increased from DEM to ESCC and from ESCS to ESCC, but there was no significant increase from DEM to ESCS (Fig. 2A). We recognized limitations of the study are small size and limited survival information for dysplasia or carcinoma in situ patients. Nevertheless, the hallmark of our findings was the Ku80 examination in resection samples, indicating the potential of Ku80 as an early tumor marker. This is the first study suggesting Ku80 upregulation in preneoplastic lesions and ESCS, which provides an indicator for early detection and intervention of ESCC.

Next, we divided 107 superficial ESCC into two groups according to the Ku80 expression level. Further analyses indicated Ku80 overexpression is closely related to low differentiation degree, invasive T status, positive nodal involvement, advanced TNM stage, and malignancy recurrence (Table 1). Our results were consistent with previous studies 18, 26, 27, which suggested Ku80 upregulation was closely related to key clinicopathological features in lung adenocarcinoma and breast cancer.

Despite the advance in diagnosis and perioperative management, the prognosis of superficial ESCC is unsatisfactory 3, 7. In present study, there were still 57 patients (56.4%) who had recurrence during 5‐year follow‐up. The 5‐year OS and DFS are only 62.4% and 45.5%, which were similar with data of the previous studies 11, 28, 29. Midthoracic superficial ESCC patients with en bloc resection should not be received postoperative adjuvant therapy, based on NCCN guideline. In our opinion, postoperative adjuvant therapy should be given to patients who are at higher probability of worse prognosis 7. The tricky problem is that how to identify the high‐risk patients. Several studies had suggested that Ku80 has significant functions and might be a biomarker in ESCC 20, 21, 22, 30, 31. Additionally, some interesting findings about the prognostic values of Ku80 in malignant cancers had been reported. Ma Q et al. 27 demonstrated Ku80 was related to survival of patients with lung adenocarcinoma. But study of Soderlund et al. 32 did not support that Ku80 could predict outcomes of breast cancer. Even in same malignant tumor, the findings were controversial 33, 34. There is no available information about the relationship between Ku80 and prognosis of superficial ESCC patients to our knowledge. Here, our data indicated long OS and DFS of midthoracic superficial ESCC patients were related to Ku80 low expression (Table 2 and 3). Additionally, multivariate analyses demonstrated Ku80 expression was an independent predictor of OS and DFS in superficial ESCC. These findings demonstrated Ku80 evaluation might provide important information about clinical outcome and management in superficial ESCC.

To understand the biological roles of Ku80, the lentiviral vectors for shRNA were used to stably suppress Ku80 expression in ESCC cells. Next, we studied the effects of Ku80 silencing on malignant behavior of ESCC cells. Figures 3 and 4 illustrated the effects of Ku80 depletion on the cell proliferation, clone formation, and apoptosis of ESCC cells in vitro. We also performed experiments in vivo to validate effectiveness of Ku80 knockdown (Fig. 4C). Tumor growth was greatly suppressed in Ku80 shRNA‐transfected cells. However, control and scramble shRNA‐infected xenograft grew aggressively. Ku80 could repair the DNA damage rapidly by rejoining broken ends irrespective of DNA sequence. Chaotic Ku80 expression could cause aberrant DNA damage reaction. Normal DNA damage reaction can remove DNA repair and reduce genome instability 35. However, imprecise repair of DNA induces genomic instability and gene mutations 36. Abnormal activation of Ku80 is a feature of ESCC cells, which results in cellular microenvironment predisposed to degeneration and carcinogenesis. Lentiviral‐based shRNA might be utilized as an effective cancer therapy 37. Our study provided a promising strategy for gene therapy in ESCC. We demonstrated the potency of Ku80 silencing in inhibition of cell proliferation and induction of apoptosis. Additionally, the blockage of tumorigenesis by lentiviral‐mediated Ku80 shRNA also supported the effectiveness of this strategy in ESCC.

In addition, we recognized limitations of the study. Replication studies with larger sample size are necessary to validate clinical significance of Ku80 in ESCC. Other DNA repair proteins are involved in development of malignant disease 38, 39. Moreover, Ku70 and Ku80 form a heterodimer, Ku protein, which could bind to the DNA ends and act as a DNA repair protein 40. So tissue microarray detection of superficial ESCC is needed to further illuminate the function of NHEJ DNA repair proteins in carcinogenesis of ESCC. Another limitation of the study is that we did not have mechanistic research. Further studies are needed to clarify the mechanism of biological functions of Ku80 in ESCC.

In conclusion, this study provided evidence that Ku80 had unrecognized roles in carcinogenesis and development of ESCC. Ku80 might serve as an early diagnostic biomarker for dysplasia, carcinoma in situ, and superficial ESCC. We also demonstrated that Ku80 could be used as an independent predictor in superficial ESCC. Superficial ESCC cannot always be successfully treated by the radical resection, and some patients would experience postoperative recurrent disease. We provide a new method to predict recurrence risk and survival of these patients. Furthermore, our findings in vitro and in vivo suggested that Ku80 silencing could inhibit malignant behavior of ESCC. Therefore, Ku80 could be exploited as a new therapeutic target for ESCC.

## Conflict of Interest

The authors declare that they have no conflict of interests..
